# Associations between high cholesterol and insulin sensitivity in diabetic versus non-diabetic among young adults in Lephalale, Limpopo Province, South Africa

**DOI:** 10.3389/fendo.2025.1650989

**Published:** 2025-09-23

**Authors:** Themba Titus Sigudu, Thandiwe Ntomfuthi Mkhatshwa, Kotsedi Daniel Monyeki, Moloko Matshipi

**Affiliations:** ^1^ Department of Physiology and Environmental Health, School of Molecular and Life Sciences, Faculty of Science and Agriculture, University of Limpopo, Sovenga, Limpopo, South Africa; ^2^ Division of Health and Society, School of Public Health, Faculty of Health Sciences, University of the Witwatersrand, Johannesburg, Gauteng, South Africa

**Keywords:** dyslipidemia, insulin resistance, young adults, type 2 diabetes, South Africa

## Abstract

**Background:**

The rising global burden of diabetes mellitus and associated metabolic disorders disproportionately affects low and middle-income countries, with dyslipidemia being a key contributor to cardiovascular risk in insulin-resistant individuals. Limited data exist on population-specific associations between cholesterol levels and insulin sensitivity among young adults in transitioning African communities.

**Methods:**

This cross-sectional study analyzed 781 young adults (18–29 years) from the Ellisras Longitudinal Study in Lephalale, South Africa. Participants were stratified by diabetic status using ADA criteria. Fasting blood samples assessed glucose, insulin (HOMA-IR), and lipid profiles. Multivariable linear regression evaluated cholesterol-HOMA-IR associations, adjusted for confounders.

**Results:**

Diabetics (n=169) showed significantly higher total cholesterol (5.1 vs. 4.3 mmol/L), LDL-C (3.2 vs. 2.6 mmol/L), triglycerides (1.8 vs. 1.1 mmol/L), and lower HDL-C (1.0 vs. 1.2 mmol/L) than non-diabetics (all p<0.001). Dyslipidemia prevalence was 2–3 times higher in diabetics (e.g., 52.7% vs. 23.2% for high total cholesterol). HOMA-IR correlated positively with total cholesterol (β=0.42, p<0.001), LDL-C (β=0.38, p<0.001), and triglycerides (β=0.47, p<0.001), and inversely with HDL-C (β=−0.51, p<0.001).

**Conclusion:**

Young diabetic adults in Lephalale exhibit pronounced dyslipidemia and insulin resistance, with strong lipid-HOMA-IR associations. Findings highlight the need for early metabolic screening and targeted interventions in transitioning African communities to mitigate future cardio metabolic risk.

## Introduction

The global burden of diabetes mellitus (DM) and associated metabolic disorders continues to rise at an alarming rate, with type 2 diabetes (T2DM) accounting for more than 90% of cases worldwide (1). The International Diabetes Federation (IDF) estimates that 537 million adults were living with diabetes in 2021, a number projected to increase to 783 million by 2045, with low- and middle-income countries (LMICs) bearing the greatest burden (2). Dyslipidemia, particularly elevated cholesterol levels, is a common metabolic abnormality observed in individuals with impaired insulin sensitivity and is strongly associated with increased cardiovascular disease (CVD) risk (3, 4). Insulin resistance disrupts lipid homeostasis, leading to atherogenic dyslipidemia, a condition characterized by elevated triglycerides, increased small dense LDL-C particles, and reduced HDL-C (5). Although the link between insulin resistance and abnormal lipid metabolism is well established, population-specific analyses, particularly among young adults in rural or transitioning communities, remain limited, particularly in sub-Saharan Africa (SSA), where epidemiological data on metabolic risk factors are scarce (6, 7).Insulin sensitivity plays a central role in the regulation of glucose and lipid metabolism. Reduced insulin sensitivity (insulin resistance) has been associated with higher total cholesterol, LDL-C, and triglyceride levels, while concurrently lowering HDL-C (8). This dysregulation accelerates the progression of atherosclerosis, coronary artery disease, and other cardiometabolic complications, particularly in individuals with pre-existing diabetes (9, 10). Studies suggest that insulin resistance precedes overt diabetes by several years, and early metabolic disturbances in lipid profiles may serve as predictive biomarkers for future cardiometabolic disease (11). However, the extent to which these associations manifest in younger populations, and whether differences exist between diabetic and non-diabetic individuals within the same socio-geographic context, requires further investigation, particularly in African settings where genetic and environmental influences may modulate metabolic risk (12).

Lephalale, located in Limpopo Province, South Africa, is a region undergoing rapid urbanisation and industrial development, largely driven by coal mining and power generation (13). These socioeconomic transitions have influenced lifestyle behaviours, including shifts towards energy-dense diets, reduced physical activity, and increased sedentary behaviour, contributing to an emerging epidemic of non-communicable diseases (NCDs) such as diabetes, dyslipidemia, and obesity among younger populations (14, 15). Despite these trends, data on metabolic health indicators in this region remain scarce, and few studies have explored the interaction between cholesterol levels and insulin sensitivity within different glycaemic states (normoglycemic, prediabetic, and diabetic) in young adults (16). Given that early metabolic dysfunction may be reversible with lifestyle or pharmacological interventions, identifying at-risk individuals before the onset of overt disease is crucial for preventive healthcare strategies (17).

This study therefore aims to investigate the associations between high cholesterol and insulin sensitivity among diabetic and non-diabetic young adults in Lephalale, using data from the Ellisras Longitudinal Study (ELS) cohort. By stratifying the analysis by diabetic status, we seek to identify potential early metabolic differences that may inform targeted intervention and prevention strategies tailored to the unique health needs of young South Africans. Understanding these relationships could contribute to early risk stratification and public health policies aimed at curbing the rising burden of metabolic diseases in transitioning African communities (18).

## Materials and methods

### Study design and setting

This cross-sectional study was conducted using data collected from community health screenings in Lephalale, Limpopo Province, South Africa. Lephalale is a rapidly urbanising region influenced by industrial and socio-economic transitions, making it an ideal setting to explore emerging non-communicable disease trends. The study was designed to examine metabolic risk factors, particularly the relationship between cholesterol levels and insulin sensitivity among young adults with differing diabetic statuses.

### Study population

A total of 781 participants were enrolled in the study, consisting of 376 males and 405 females aged between 18 and 29 years. Participants were recruited through convenience sampling during community-based health screening campaigns held across various sites within Lephalale. Inclusion criteria were: age 18–29 years, availability of complete fasting glucose, insulin, and lipid profile data. Exclusion criteria included pregnancy, history of chronic disease other than diabetes, or current use of lipid-lowering or glucose-lowering medication not related to a diabetes diagnosis.

### Classification of diabetic status

Participants were classified as diabetic or non-diabetic based on the American Diabetes Association (ADA) diagnostic criteria (1). Individuals were considered diabetic if they had any of the following: fasting plasma glucose ≥7.0 mmol/L, 2-hour post-load glucose ≥11.1 mmol/L, HbA1c ≥6.5%, or self-reported physician diagnosis of diabetes. Participants with fasting plasma glucose <5.6 mmol/L and HbA1c <5.7%, with no prior diagnosis of diabetes, were categorised as non-diabetic.

### Biochemical measurements

Fasting venous blood samples were collected from all participants after a minimum of 8 hours of overnight fasting. Trained healthcare professionals performed the blood draws during community screening events. The collected samples were processed immediately to separate serum, which was then stored at –80°C until analysis. Fasting plasma glucose levels were measured using a glucose oxidase enzymatic method, ensuring high specificity and sensitivity. Fasting insulin concentrations were assessed using a chemiluminescent immunoassay, a widely validated technique for quantifying circulating insulin levels. Lipid profile parameters, including total cholesterol (TC), low-density lipoprotein cholesterol (LDL-C), high-density lipoprotein cholesterol (HDL-C), and triglycerides (TG), were analysed using standard automated enzymatic assays. All analyses were performed in accordance with the manufacturer’s guidelines and under rigorous quality control conditions to ensure data accuracy and reliability.

### Assessment of insulin sensitivity

Insulin resistance was assessed using the Homeostatic Model Assessment of Insulin Resistance (HOMA-IR), a widely used method to estimate insulin sensitivity based on fasting insulin and glucose concentrations. The HOMA-IR value was calculated using the following formula:


HOMA−IR=(Fasting Insulin(μU/mL)×Fasting Glucose(mmol/L))/22.5

This model assumes a feedback loop between hepatic glucose production and insulin secretion under fasting conditions, with higher HOMA-IR scores indicating lower insulin sensitivity (i.e., greater insulin resistance). Fasting blood samples were collected after an overnight fast of at least 8–10 hours, and plasma glucose and insulin levels were measured using standardized biochemical assays.

For the purpose of subgroup analyses and clinical interpretation, HOMA-IR values were also categorized based on established cut-off points derived from the literature to define the presence of insulin resistance. Participants with HOMA-IR values above the cut-off threshold were classified as insulin-resistant, while those below were considered insulin-sensitive. This approach enabled both continuous and categorical analyses of insulin resistance within the study population, thereby enhancing the robustness and clinical relevance of the findings.

### Statistical analysis

All statistical analyses were performed using STATA version 18 (StataCorp LLC, College Station, TX, 2023). Descriptive statistics were presented as means and standard deviations for continuous variables and as frequencies and percentages for categorical variables. Independent sample t-tests and chi-square tests were used to compare variables between diabetic and non-diabetic groups.

Multivariable linear regression models were constructed to assess the association between cholesterol levels and insulin resistance (HOMA-IR), adjusting for potential confounders including age and sex. Interaction terms were included to evaluate effect modification by diabetic status. Statistical significance was set at p < 0.05.

### Ethical considerations

Ethical approval for the study was granted by the Turfloop Research Ethics Committee (MREC/P/204/2013: IR and TREC/323/2017: IR-Renewed). Written informed consent for participation was not required from the participants or their legal guardians/next of kin in accordance with South African national research regulations and the ethics committee’s approved protocol, as data were collected during routine community health screenings. However, verbal informed consent was obtained from all participants prior to data collection, and participation was entirely voluntary.

## Results

### Descriptive statistics

#### Characteristics of study participants

**Table 1** presents the demographic and clinical characteristics of the study population stratified by diabetic status. A total of 781 young adults were included, of whom 612 (78.4%) were non-diabetic and 169 (21.6%) were diabetic. The overall mean age of the cohort was 24.5 ± 3.8 years. Diabetic participants were significantly older than their non-diabetic counterparts (26.1 ± 4.2 vs. 23.9 ± 3.6 years, p < 0.001).

**Table 1:** Baseline characteristics of study participants.

Characteristics	Total	Non-diabetic	Diabetic	P-value
	(n=781)	(n=612)	(n=169)	
Age (years)	24.5 ± 3.8	23.9 ± 3.6	26.1 ± 4.2	<0.001
Sex (Male), n (%)	376 (48.1%)	298 (48.7%)	78 (46.2%)	0.562
Sex (Female), n (%)	405 (51.9%)	314 (51.3%)	91 (53.8%)	0.619
SBP (mmHg)	122 ± 14	119 ± 12	130 ± 16	<0.001
DBP (mmHg)	76 ± 10	74 ± 9	81 ± 11	<0.001

Data presented as mean ± SD or n (%). p-values from t-tests (continuous) or chi-square tests (categorical).

Sex distribution did not differ significantly between the two groups. Males constituted 48.1% of the overall sample, with comparable proportions in the non-diabetic (48.7%) and diabetic (46.2%) groups (p = 0.562). Similarly, females accounted for 51.9% of the total sample, with no significant difference between non-diabetic (51.3%) and diabetic (53.8%) participants (p = 0.619).

Blood pressure levels were notably higher among diabetic individuals. Mean systolic blood pressure (SBP) was significantly elevated in the diabetic group compared to the non-diabetic group (130 ± 16 mmHg vs. 119 ± 12 mmHg, p < 0.001). Likewise, mean diastolic blood pressure (DBP) was higher in diabetics (81 ± 11 mmHg) than in non-diabetics (74 ± 9 mmHg), with the difference reaching statistical significance (p < 0.001).

#### Biochemical and metabolic profiles by diabetic status

Significant differences were observed in the biochemical and metabolic parameters between diabetic and non-diabetic participants (**Table 2**). Diabetic individuals exhibited markedly elevated fasting plasma glucose (FPG) levels (8.2 ± 2.1 mmol/L) compared to non-diabetics (4.9 ± 0.5 mmol/L), with a highly significant p-value (<0.001). Similarly, glycated haemoglobin (HbA1c) was significantly higher among diabetics (7.6 ± 1.5%) than non-diabetics (5.2 ± 0.3%), reflecting poorer long-term glycaemic control (p < 0.001).

**Table 2:** Biochemical and metabolic profiles by diabetic status.

Parameter	Non-diabetic (n=612)	Diabetic (n=169)	P-value
FPG^1^ (mmol/L)	4.9 ± 0.5	8.2 ± 2.1	<0.001
HbA1c (%)	5.2 ± 0.3	7.6 ± 1.5	<0.001
Fasting Insulin (µU/mL)	8.5 ± 3.2	14.7 ± 6.8	<0.001
HOMA-IR^2^	1.9 ± 0.8	5.4 ± 2.6	<0.001
Total Cholesterol (mmol/L)	4.3 ± 1.0	5.1 ± 1.3	<0.001
LDL-C (mmol/L)	2.6 ± 0.8	3.2 ± 1.1	<0.001
HDL-C (mmol/L)	1.2 ± 0.3	1.0 ± 0.3	<0.001
Triglycerides (mmol/L)	1.1 ± 0.6	1.8 ± 0.9	<0.001

Data are presented as mean ± SD. p-values calculated using independent t-tests. ^1^FPG: Fasting Plasma Glucose; ^2^HOMA-IR: Homeostatic Model Assessment of Insulin Resistance.

Insulin resistance, as assessed by fasting insulin and HOMA-IR, was also substantially elevated in the diabetic group. Diabetics had a mean fasting insulin level of 14.7 ± 6.8 µU/mL compared to 8.5 ± 3.2 µU/mL in non-diabetics (p < 0.001), while HOMA-IR values were significantly higher in diabetics (5.4 ± 2.6) versus non-diabetics (1.9 ± 0.8) (p < 0.001), indicating greater insulin resistance among those with diabetes.

Lipid profiles also differed significantly by diabetic status. Diabetic participants had higher total cholesterol (5.1 ± 1.3 mmol/L vs. 4.3 ± 1.0 mmol/L, p < 0.001) and LDL-C levels (3.2 ± 1.1 mmol/L vs. 2.6 ± 0.8 mmol/L, p < 0.001), along with significantly lower HDL-C concentrations (1.0 ± 0.3 mmol/L vs. 1.2 ± 0.3 mmol/L, p < 0.001). Triglyceride levels were also elevated in the diabetic group (1.8 ± 0.9 mmol/L) compared to non-diabetics (1.1 ± 0.6 mmol/L), with a p-value < 0.001.

#### Prevalence of dyslipidemia by diabetic status

The prevalence of dyslipidemia was significantly higher among diabetic participants compared to their non-diabetic counterparts across all lipid parameters (**Table 3**). High total cholesterol (TC ≥ 5.2 mmol/L) was observed in 52.7% of diabetics, more than double the prevalence in non-diabetics (23.2%) (p < 0.001). Similarly, elevated low-density lipoprotein cholesterol (LDL-C ≥ 3.4 mmol/L) was present in 42.6% of diabetics versus 16.0% of non-diabetics (p < 0.001), indicating a substantially greater atherogenic lipid burden in the diabetic group.

**Table 3:** Prevalence of dyslipidemia by diabetic status.

Dyslipidemia parameter	Non-diabetic (n=612)	Diabetic (n=169)	P-value
High TC^1^ (≥5.2 mmol/L)	142 (23.2%)	89 (52.7%)	<0.001
High LDL-C^2^ (≥3.4 mmol/L)	98 (16.0%)	72 (42.6%)	<0.001
Low HDL-C^3^ (<1.0 M, <1.3 F)	156 (25.5%)	94 (55.6%)	<0.001
High TG^4^ (≥1.7 mmol/L)	112 (18.3%)	87 (51.5%)	<0.001

^1^TC, Total Cholesterol; ^2^LDL-C, Low-Density Lipoprotein Cholesterol; ^3^HDL-C, High-Density Lipoprotein Cholesterol; ^4^TG, Triglycerides.

Low high-density lipoprotein cholesterol (HDL-C <1.0 mmol/L for males, <1.3 mmol/L for females), a known cardiovascular risk factor, affected over half (55.6%) of the diabetic participants, compared to 25.5% of non-diabetics (p < 0.001). In addition, high triglyceride levels (TG ≥ 1.7 mmol/L) were significantly more prevalent among diabetics (51.5%) than non-diabetics (18.3%) (p < 0.001).

### Inferential statistics

#### Multivariable linear regression

##### Association between lipid parameters and insulin resistance

Multivariable linear regression analysis revealed significant associations between lipid parameters and insulin resistance, as measured by HOMA-IR, after adjusting for age and sex. (**Table 4**). Total cholesterol was positively associated with HOMA-IR (β = 0.42, 95% CI: 0.31–0.53, p < 0.001), indicating that for every 1 mmol/L increase in total cholesterol, there was a corresponding increase in insulin resistance. Similarly, LDL-C showed a significant positive association (β = 0.38, 95% CI: 0.27–0.49, p < 0.001), supporting its role in impaired insulin sensitivity.

**Table 4:** Multivariable linear regression of HOMA-IR and cholesterol levels.

Variable	β (95% CI)	P-value	Adjusted r²
Total Cholesterol (per 1 mmol/L)	0.42 (0.31–0.53)	<0.001	0.28
LDL-C (per 1 mmol/L)	0.38 (0.27–0.49)	<0.001	0.25
HDL-C (per 1 mmol/L)	-0.51 (-0.63–0.39)	<0.001	0.31
TG (per 1 mmol/L)	0.47 (0.35–0.59)	<0.001	0.29

Adjusted for age and sex.

In contrast, HDL-C was inversely associated with HOMA-IR (β = –0.51, 95% CI: –0.63 to –0.39, p < 0.001), suggesting a protective effect of higher HDL-C levels against insulin resistance. Triglycerides also demonstrated a strong positive relationship with HOMA-IR (β = 0.47, 95% CI: 0.35–0.59, p < 0.001), consistent with their known role in the pathophysiology of insulin resistance. The highest proportion of variance explained was observed for HDL-C (Adjusted R² = 0.31), followed closely by triglycerides (R² = 0.29), total cholesterol (R² = 0.28), and LDL-C (R² = 0.25).

## Discussion

The present study provides robust evidence of significant associations between lipid abnormalities and insulin sensitivity among young adults in Lephalale, Limpopo Province, with clear distinctions between diabetic and non-diabetic individuals. Our findings highlight the early emergence of dyslipidemia and insulin resistance in a transitioning rural South African population and contribute to the growing body of literature on metabolic risk profiling in sub-Saharan Africa (SSA).

Consistent with global and regional data, our results show that young adults with diabetes exhibit significantly higher levels of total cholesterol, LDL-C, and triglycerides, alongside reduced HDL-C levels, compared to their non-diabetic counterparts (19). These lipid abnormalities were paralleled by elevated fasting insulin levels and HOMA-IR scores, indicating profound insulin resistance in diabetic participants (20). These findings align with previous studies suggesting that insulin resistance is closely linked to an atherogenic lipid profile, contributing to the early pathogenesis of cardiovascular disease (CVD) in diabetic populations (21).

Importantly, multivariable regression analysis revealed strong positive associations between HOMA-IR and both total cholesterol and LDL-C, even after adjusting for potential confounders such as age and sex (22).

These findings support existing evidence of a strong association between dyslipidemia and insulin resistance, though the directionality of this relationship cannot be determined from cross-sectional data. Given the cross-sectional nature of the study, all observed relationships should be interpreted as associations, not as evidence of causation (23, 24). Conversely, HDL-C exhibited a significant inverse relationship with insulin resistance, supporting its protective metabolic role and emphasizing its relevance as a therapeutic target in young adults at risk for type 2 diabetes mellitus (T2DM) (25).

The observed prevalence of dyslipidemia was alarmingly high, particularly among diabetic participants, with more than half displaying elevated total cholesterol and low HDL-C levels (26). These proportions exceed those reported in national estimates and may reflect the impact of rapid urbanisation and lifestyle transitions unique to Lephalale’s semi-rural setting (25). Increasing access to processed foods, reduced physical activity, and sedentary occupations linked to industrial development may be contributing factors, as has been noted in other parts of SSA undergoing similar socioeconomic transitions (27, 28).

Notably, these metabolic disturbances were observed in relatively young adults, suggesting that a substantial portion of metabolic deterioration occurs early in the life course (29). This finding supports the “developmental origins of health and disease” (DOHaD) hypothesis, which posits that early life exposures, whether nutritional, environmental, or behavioural, predispose individuals to future metabolic disorders (30, 31). Given that insulin resistance and dyslipidemia are modifiable risk factors, these results highlight the importance of early screening and targeted interventions for young adults in high-risk settings.

Another critical implication of this study is the utility of HOMA-IR as a practical marker of insulin sensitivity in resource-limited settings. While not without limitations, HOMA-IR offers an accessible alternative to more invasive and costly gold-standard techniques (e.g., euglycemic clamps), especially in large-scale epidemiological surveillance of rural African populations (32). Our findings suggest that routine lipid screening, in conjunction with fasting glucose and insulin measurements, could serve as an efficient strategy for early risk stratification in primary care and community health programs.

Furthermore, the high prevalence of dyslipidemia and insulin resistance in non-diabetic individuals, although lower than in diabetics, signals an urgent need for preventive strategies that address the metabolic continuum rather than focusing solely on established diabetes. These individuals may represent a “metabolically at-risk” phenotype, which, if unaddressed, may progress to overt T2DM and cardiovascular complications over time (33, 34). Thus, integrating metabolic screening into routine health checks for young adults, particularly in rapidly urbanising areas such as Lephalale, could significantly reduce future NCD burden.

Given the high prevalence of dyslipidemia and insulin resistance observed in this study, we recommend the implementation of context-appropriate metabolic screening programs, particularly targeting adults in rural and semi-urban communities in South Africa. Integrating routine lipid and glucose assessments into existing primary healthcare services could facilitate early identification of at-risk individuals. Furthermore, public health interventions promoting healthy diets, physical activity, and awareness of cardiometabolic risk should be prioritized in these settings, where access to specialized care may be limited.

This study also fills an important gap in the literature by providing localized data from a relatively under-researched region of South Africa. Previous studies from other SSA countries have reported similar metabolic trends but often focus on older, urbanized populations (35). Our findings extend this work to a younger, semi-rural cohort, reinforcing the notion that early metabolic dysfunction is not limited to affluent or urban settings. This highlights the need for geographically tailored public health strategies that consider the unique epidemiological, cultural, and socioeconomic contexts of communities such as Lephalale. However, as a cross-sectional study, the findings cannot establish causality between dyslipidemia and insulin resistance. The observed associations may be influenced by unmeasured confounding variables or subject to reverse causation. To better understand the directionality and underlying mechanisms of these metabolic interactions, future research should employ longitudinal designs that track changes over time and explore causal pathways.

## Conclusions

This study provides compelling evidence of a significant association between elevated cholesterol levels and reduced insulin sensitivity among young adults in Lephalale, Limpopo Province, with more pronounced metabolic disturbances observed in individuals with diabetes. Diabetic participants exhibited markedly higher levels of total cholesterol, LDL-C, and triglycerides, along with significantly lower HDL-C concentrations compared to their non-diabetic counterparts. These lipid abnormalities were strongly associated with increased insulin resistance, as reflected by higher HOMA-IR values. The findings highlight the synergistic burden of dyslipidemia and insulin resistance in the early pathogenesis of cardiometabolic disorders, particularly in resource-limited and transitioning communities.

The data also highlight the early onset of metabolic dysfunction in a relatively young population, indicating the presence of cardiovascular and diabetes-related risk factors well before the development of overt clinical complications. Notably, the strong inverse association between HDL-C and insulin resistance supports its potential role as a protective marker, while elevated triglycerides and LDL-C reinforce their deleterious impact on metabolic health. These relationships persisted even after controlling for key confounders, suggesting intrinsic metabolic interactions rather than purely lifestyle-related associations.

Given the context of rapid urbanisation and lifestyle transitions in Lephalale, these findings highlight the urgent need for proactive, targeted public health strategies focusing on early screening, lifestyle modification, and education to prevent progression to overt diabetes and cardiovascular disease. Community-based interventions tailored to the unique socioeconomic and cultural dynamics of rural South African youth are critical. Furthermore, longitudinal research is warranted to explore causal relationships, track metabolic trajectories, and evaluate the long-term impact of dyslipidemia on insulin sensitivity and overall health outcomes.

Finally, this study contributes to the growing body of evidence on the metabolic risks facing young adults in sub-Saharan Africa and advocates for integrated preventive measures to curb the rising tide of non-communicable diseases in underserved regions. Early identification and intervention in high-risk individuals, even before clinical manifestation of disease, can yield substantial public health benefits and reduce future healthcare burdens.

### Strengths and limitations

One of the key strengths of this study is its focus on a vulnerable and underrepresented population, young adults in Lephalale, a rural area in Limpopo Province, South Africa. By targeting this group, the study provides valuable insights into the metabolic health of individuals often overlooked in epidemiological research. The inclusion of both diabetic and non-diabetic participants enabled meaningful comparisons of lipid profiles and insulin sensitivity across different glycaemic statuses. The use of standardised biochemical markers, including total cholesterol, LDL-C, HDL-C, triglycerides, and HOMA-IR, enhances the reliability and comparability of the findings, adding robustness to the study’s conclusions. Importantly, the data generated hold significant public health relevance, with potential to inform early intervention and screening efforts aimed at curbing the growing burden of non-communicable diseases in rural South African settings.

However, several limitations must be acknowledged. The cross-sectional design inherently limits causal inference, as it only captures associations at a single point in time. The relatively small sample size may reduce statistical power and restrict the generalisability of the findings beyond the immediate study population. Furthermore, residual confounding cannot be excluded, as factors such as dietary intake, physical activity, genetic predisposition, and socioeconomic status were not fully assessed. The reliance on self-reported diabetes status, without confirmatory diagnostic testing for all participants, may have introduced misclassification bias. Although all individuals were tested for HbA1c and fasting glucose, a small subset of diabetes classifications was based solely on self-reported physician diagnosis, raising the possibility that undiagnosed individuals or inaccurate self-reports may have skewed the results. Additionally, the use of single time-point measurements for biochemical markers may not fully reflect long-term metabolic status or variability in insulin sensitivity and lipid levels. Future research would benefit from longitudinal designs, repeated measures, and confirmatory diagnostic procedures to strengthen the validity and applicability of findings in similar underserved populations.

## Data Availability

The raw data supporting the conclusions of this article will be made available by the authors, without undue reservation.
